# Phytochemical Profiling and Green Synthesis of Silver Nanoparticles from *Quercus robur* Acorn: Characterization and Biological Evaluation

**DOI:** 10.3390/molecules31101653

**Published:** 2026-05-14

**Authors:** Mürüvvet Kurt, Serdar Güngör, Gülderen Uysal Akkuş, Atilla Evcin, Safiye Elif Korcan

**Affiliations:** 1 Department of Chemistry, Afyon Kocatepe University, Afyonkarahisar 03200, Türkiye; muruvvetkurt@aku.edu.tr (M.K.); guakkus@aku.edu.tr (G.U.A.); 2 Department of Medical Microbiology, Faculty of Medicine, Bilecik Şeyh Edebali University, Bilecik 11210, Türkiye; 3 Department of Materials Science and Engineering, Afyon Kocatepe University, Afyonkarahisar 03200, Türkiye; evcin@aku.edu.tr; 4 Vocational School of Health Services, Uşak University, Uşak 64100, Türkiye; elif.korcan@usak.edu.tr

**Keywords:** green synthesis, silver nanoparticle, antioxidant capacity, antimicrobial activity, acorn, essential oils, biological evaluation

## Abstract

Silver nanoparticles (AgNPs) were synthesized via a green approach using aqueous extract of *Quercus robur* acorn as a reducing and stabilizing agent. The synthesis process was optimized at 1 mM Ag^+^ concentration, yielding stable nanoparticles with a characteristic surface plasmon resonance peak at 445 nm. Structural and morphological analyses confirmed the formation of predominantly spherical nanoparticles with particle sizes ranging between 40 and 68 nm and a face-centered cubic crystalline structure. Phytochemical analyses revealed significantly higher total phenolic and total flavonoid contents in the crude acorn extract compared to the synthesized nanoparticles, indicating the involvement of these compounds in the phytoreduction process. Although antioxidant activity decreased after nanoparticle formation, phyto-mediated AgNPs (PAgNPs) exhibited notable antibacterial activity, particularly against *Staphylococcus aureus* with a minimum inhibitory concentration of 26 µg/mL. Antibiotic combination assays demonstrated additive and synergistic interactions depending on the tested microorganism. Gas Chromatography–Mass Spectrometry (GC–MS) analysis of acorn essential oil identified β-caryophyllene (43.1%) as the major component, suggesting the presence of bioactive terpenoids potentially contributing to nanoparticle stabilization. These findings demonstrate that *Quercus robur* acorn extract can serve as suitable phytogenic source for the controlled synthesis of silver nanoparticles with moderate antibacterial potential.

## 1. Introduction

Nanoparticles (NPs), particularly metal-based nanomaterials such as silver nanoparticles (AgNPs), have attracted significant attention due to their unique physicochemical properties, including catalytic activity, optical behavior and broad-spectrum antimicrobial effects [1]. Conventional chemical and physical synthesis routes often involve toxic reducing and stabilizing agents, which may limit their biomedical applicability [2]. In contrast, green synthesis approaches using plant-derived biomolecules provide an eco-friendly and biocompatible alternative [3]. Plant-mediated biosynthesis of metallic nanoparticles occurs through phytochemicals containing functional groups capable of reducing metal ions [4]. Compounds such as phenolics, flavonoids, terpenoids, proteins and alkaloids can act as both reducing and capping agents during nanoparticle formation [5,6,7].

The reduction mechanism is mainly attributed to biomolecules such as terpenoids, flavones, tannins and other phenolic compounds naturally present in plant biomass [8,9]. The use of water as a solvent further enhances the environmental compatibility of the synthesis process [10].

*Quercus robur*, commonly known as pedunculate oak, belongs to the Fagaceae family and is widely distributed in Turkey [11]. Acorns are particularly rich in tannins, phenolic compounds and flavonoids, which are associated with strong antioxidant and antimicrobial properties [12,13]. Tannins have been reported to inhibit bacterial growth by interacting with metal ions and bacterial enzymes [14,15].

Although silver nanoparticles have been successfully synthesized previously using bark extracts of various *Quercus* species, the phytochemical composition of acorns differs significantly from that of the bark. Acorns are particularly rich in volatile terpenoids, phenolic acids, and hydrolyzable tannins. To our knowledge, no previous studies have investigated: (i) the volatile oil profile of *Quercus robur* acorn in the context of nanoparticle synthesis, (ii) different aqueous extraction methods (Soxhlet, boiling, and room temperature) for acorn-mediated AgNPs production, or (iii) the synergistic potential of acorn-derived AgNPs with conventional antibiotics. Therefore, the present study aims to (i) synthesize silver nanoparticles using *Quercus robur* acorn extract via a green synthesis approach, (ii) characterize their physicochemical properties using multiple analytical techniques, (iii) evaluate their antioxidant and antibacterial activities, and (iv) investigate their interactions with conventional antibiotics.

The scientific novelty of this study lies in the use of acorn as a phytochemically distinct plant matrix compared to commonly studied bark extracts, together with the combined assessment of extraction methods and antibiotic interaction effects. This approach provides new insights into the role of acorn-derived biomolecules in nanoparticle formation and their contribution to biological activity. This approach is scientifically important for understanding how different plant matrices influence nanoparticle synthesis mechanisms and functional properties.

## 2. Results and Discussion

### 2.1. Green Synthesis of Silver Nanoparticles

According to the LaMer model, AgNPs formation proceeds through nucleation followed by particle growth [16]. In this study, different Ag^+^ concentrations (0.1, 1 and 5 mM) were evaluated to determine optimal synthesis conditions. Homogeneous nanoparticle formation was observed only at 1 mM AgNO_3_, while no stable nanoparticles formed at other concentrations. This indicates that an appropriate Ag^+^ concentration is required to overcome the nucleation energy barrier and ensure controlled particle formation.

### 2.2. Acorn Essential Oil Extraction and Essential Oil Analysis with GC-MS

According to the *Quercus robur* acorn essential oil analysis results, 27 compounds were detected and constituted 95.26% of the total compounds detected (Table 1). It appears that β-caryophyllene is the most abundant component in *Quercus robur* acorn essential oil with a rate of 43.1%. This is followed by α-pinene with 21.1% and α-humulene with 8.2%.

The abundance of sesquiterpenes such as β-caryophyllene suggests possible contribution to nanoparticle stabilization through hydrophobic surface interactions. Although nanoparticle synthesis was carried out using aqueous extract, volatile terpenoid compounds identified in the essential oil may indirectly contribute to nanoparticle stabilization or biological activity through associated phytochemical interactions.

### 2.3. Determination of Total Phenolic Substance and Total Flavonoid Substance by Folin-Ciocalteu Method

**Figure 1:**
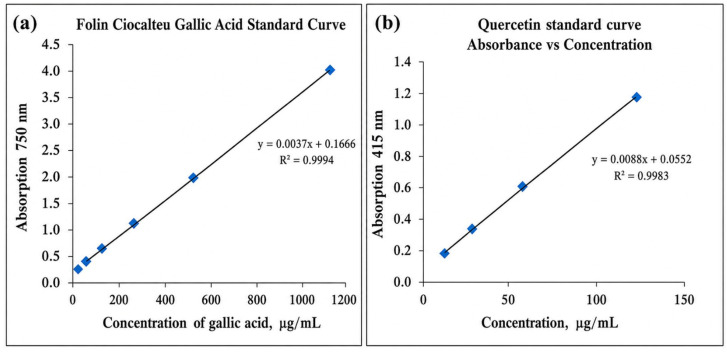
(**a**) Gallic acid calibration curve graph (**b**) Quercetin calibration curve graph. Blue diamond symbols represent experimental data points.

Total phenolic and flavonoid contents in the crude extract and nanoparticles were measured to confirm their roles in the phytoreduction process. Phenolics and flavonoids are nucleophilic due to the presence of aromatic rings, thus responsible for having chelating potential [17]. The total phenolic substance amounts of the samples were determined as mg Gallic Acid Equivalents (GAE)/g with the help of the equation obtained from the standard chart prepared using different concentrations of gallic acid solution (A = 0.0037c + 0.1666, r2 = 0.99, n = 3). (Figure 1a). Although higher absorbance values were recorded at elevated concentrations, quantitative analysis was restricted to the linear range of the calibration curve to ensure accuracy. Quercetin was used as the standard and total flavonoid content was determined using a calibration curve (A = 0.0088c + 0.0552, r2 = 0.99, n = 3). Flavonoid contents of the samples were calculated as Quercetin equivalent (µg QE/g) (Figure 1b).

Total phenolic substance content was determined to be higher in acorn extract than in PAgNPs. While the amount of phenolic substance was found to be 136.594 ± 0.700 µg GAE/g in *Quercus robur* acorn extract, it was 112.00 ± 0.08 µg GAE/g for PAgNPs (Table 2). The decreased amount of phenolic and flavonoid compounds measured in the synthesized AgNPs indicates a reduction in antioxidant activity after synthesis, which may be associated with the involvement of these compounds in nanoparticle formation. In the study conducted by Phull et al. [18], reporting that phenolic and flavonoid compounds in silver nanoparticles synthesized using Bergenia ciliatarhizome decreased compared to the extract, supports our results. In another study, Sohal et al. [19] found that the amount of phenolic and flavonoid compounds in silver nanoparticles synthesized using aloe vera gel extract decreased compared to the extract. Our results are similar to these studies. The decreased amount of phenolic and flavonoid compounds in the synthesized AgNPs indicates their involvement in nanoparticle formation and subsequent surface capping. These biomolecules are known to possess various biological activities, including antioxidant and antimicrobial effects; however, their reduced levels after synthesis suggest that they are largely consumed or bound to the nanoparticle surface rather than remaining in free form. This reduction may therefore explain the observed decrease in antioxidant activity of the synthesized nanoparticles compared to the crude extract. In addition, flavonoids have also been reported to be highly efficient oxidant scavengers [20]. In our study, there is a decreasing order of flavonoid content between the extract and its nanoparticles: Acorn extract > PAgNPs. Total flavonoid content was found to be 9.522 µg QE/g and 7 ± 0.011 µg QE/g for acorn extract and PAgNPs, respectively.

### 2.4. Characterization of Synthesized Nanoparticles

#### 2.4.1. UV–Visible Spectroscopy Analysis

**Figure 2:**
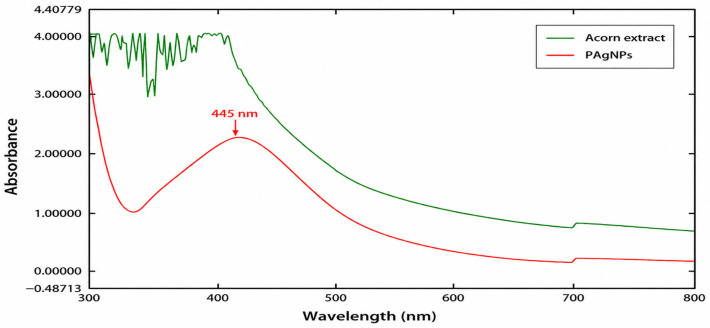
UV–Vis spectra of acorn extract and synthesized PAgNPs recorded in the wavelength range of 300–800 nm. The characteristic surface plasmon resonance (SPR) peak observed at approximately 445 nm in the PAgNPs spectrum confirms the formation of silver nanoparticles.

UV–Vis spectra of PAgNPs and *Quercus robur* acorn extract are given in Figure 2. The phytoreduction of silver ions in aqueous solutions was monitored by UV–Vis spectroscopy and confirmed the distinctive Surface Plasmon Resonance (SPR) spectrum with absorbance recorded in the range of 300–800 nm. In addition to the excitation of surface plasmon vibrations, the color of the solution changes from yellow to brown, indicating the formation of nanoparticles [21,22,23].

The formation of AgNPs was confirmed by UV–Vis spectroscopy through the appearance of a characteristic surface plasmon resonance band at 445 nm together with a visible color change from pale yellow to brown. Similar SPR bands around 420–440 nm have been reported for plant-mediated AgNPs [24,25]. The absence of this band in the plant extract indicates that nanoparticle formation occurred only after Ag^+^ reduction. (Figure 2). When the same process was repeated for the water extract of the plant, no band was detected.

#### 2.4.2. FT-IR Spectrophotometer Analysis

The presence of functional groups in the structure of the plant extract and the change in functional groups involved in reduction at the end of the reaction were examined by analysis using an FT-IR spectrophotometer in the range of 4000–400 cm^−1^.

**Figure 3:**
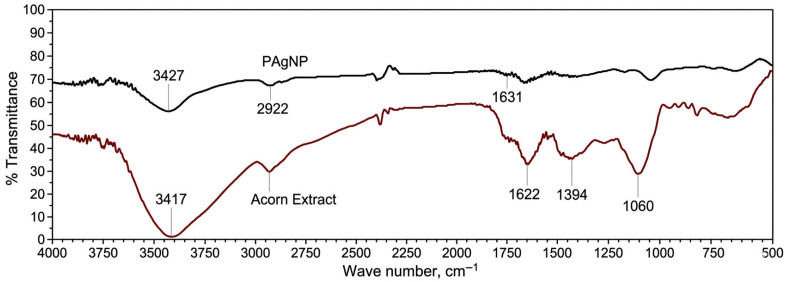
FTIR spectra of PAgNPs and Acorn extract. The black line represents acorn extract, whereas the red line represents PAgNPs.

The presence of different functional groups in *Quercus robur* acorn extract and silver nanoparticle and their binding characteristics are given in Table 3. Figure 3 shows the FT-IR spectrum of PAgNPs and acorn extract. Biomolecules and metal ions contained in plant extracts used for the synthesis of AgNPs play an important role as reductants, capping agents and stabilizers during the synthesis. In the synthesized nanoparticles, the functional groups of these biomolecules remain bound either within the structure or on the surface.

This comparison (between crude extract and PAgNPs) is primarily used to demonstrate that the acorn extract plays an active role in AgNP synthesis and that extract residues remain on the surface of the synthesized nanoparticles. The decrease in phenolic and flavonoid content indicates the consumption of these compounds during the reduction of silver ions, while the measurable amount of phenolic compounds still present in PAgNPs (112 µg GAE/g) suggests that these compounds remain on the nanoparticle surface as capping/stabilizing agents.

Filip et al. [26] compared the extract and nanoparticle peaks in the FTIR analysis of AgNPs synthesized using Cornelian cherry fruit; they determined that 3159 cm^−1^ O-H, 1717 cm^−1^ C=O, 1224 cm^−1^ C-OO groups were responsible in Ag^+1^ reduction.

Peak values of the extract obtained from *Prunus japonica* (Japanese bush cherry) fruit; were found to be 3284 cm^−1^ (N-H), 2927 cm^−1^ (C-H), 2363 cm^−1^ (C-H), 1598 cm^−1^ (C=O), 1384 cm^−1^, 1070 cm^−1^ (C-OH). When compared to the peak values of AgNPs synthesized from this extract, it has been reported that C=O, N-H and C-H groups play a role in reduction [27].

As a result, in our study, the shifts in the vibration bands of the extracts and synthesized silver nanoparticles are thought to be associated with biomolecules. By adding Ag^+^ to the extract, the bioactive compounds in the extract reacted with Ag^+^. After phytoreduction, a change in the positions and intensities of the vibration bands was observed. The change/decrease in the bands can be interpreted as the reduction in Ag^+^ ions to silver nanoparticles by some substances in the extract.

#### 2.4.3. Transmission Electron Microscopy (TEM)

**Figure 4:**
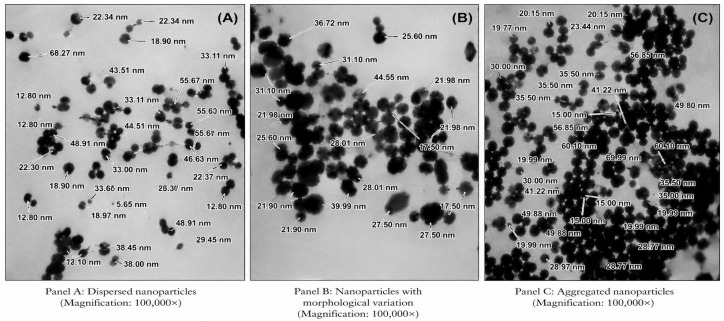
Transmission electron microscopy (TEM) images of silver nanoparticles synthesized using *Quercus robur* acorn extract: (**A**–**C**) representative micrographs showing the morphology and distribution of the nanoparticles at different regions. The nanoparticles are predominantly spherical with slight aggregation, and particle sizes were observed in the range of approximately 20–70 nm.

TEM analysis was performed to examine the morphology and approximate size distribution of the synthesized silver nanoparticles. The TEM micrographs revealed that the nanoparticles were predominantly spherical to quasi-spherical in shape with slight aggregation (Figure 4). The particle sizes were observed to fall within the nanoscale range, approximately between 20 and 70 nm.

The statistical analysis of the metallic core size distribution, derived from TEM micrographs, reveals a mean particle diameter of 29.40 pm 14.20 nm (n > 50). The particle sizes exhibit a broad distribution, ranging from a minimum of 5.65 nm to a maximum of 68.27 nm. The relatively high standard deviation indicates the polydisperse nature of the synthesized nanoparticles, which is consistent with the morphological diversity observed in the representative TEM panels.

TEM and SEM results of AgNPs synthesized from *Forsythia suspensa* (Golden bowl) fruit show that the sizes vary between 22.4 and 35.3 nm and their shapes are spherical [28]. In TEM analysis of nanoparticles synthesized using garlic extract, their shapes were spherical and their sizes were in the range of 10–50 nm [29]. In the TEM analysis of nanoparticles obtained by green synthesis from blackberries, it was reported that their shapes were spherical and their sizes were between 12 and 50 nm [30]. It is evident that the sizes and shapes of AgNPs obtained in the studies are compatible with the results obtained in our study.

The thin organic layer observed on the surface of the nanoparticles consists of phytochemical residues derived from *Quercus robur* acorn extract. While this organic layer plays a significant role as a capping and stabilizing agent for the nanoparticles, the hydrophobic interactions of the nanoparticles in TEM—due to hydrogen bonds, van der Waals forces, and hydrophobic interactions formed between the surface-bound organic molecules—cause them to appear adhered, overlapping, or clustered together. This type of partial aggregation is commonly observed in plant-mediated green synthesis and does not necessarily imply instability; rather, it reflects the presence of a biomolecular layer on the nanoparticle surface. TEM images revealed that small particle aggregates were coated with a thin organic layer that acted as a capping agent. This can also explain the fact that nanoparticles show a very good dispersion in bioreduced aqueous solution, even on a macroscopic scale [31].

As shown in Figure 4, the TEM micrographs revealed partial aggregation of the PAgNPs. This aggregation is consistent with the brownish-yellow color of the colloidal solution. The observed aggregation may be attributed to two main factors: (i) insufficient capping/stabilization by the acorn extract phytochemicals during the reduction process, and (ii) possible particle clustering during the drying step prior to TEM analysis. Despite this partial aggregation, the UV–Vis spectrum (Figure 2) displayed a single and relatively single SPR peak at 445 nm, indicating that aggregation was not extensive. Nevertheless, this partial aggregation is acknowledged as a limitation of the present study, and future optimization of synthesis conditions (e.g., pH, temperature, or the use of additional stabilizers) may improve colloidal stability.

#### 2.4.4. Scanning Electron Microscopy Energy Dispersive X-Ray Spectroscopy (SEM-EDX)

**Figure 5:**
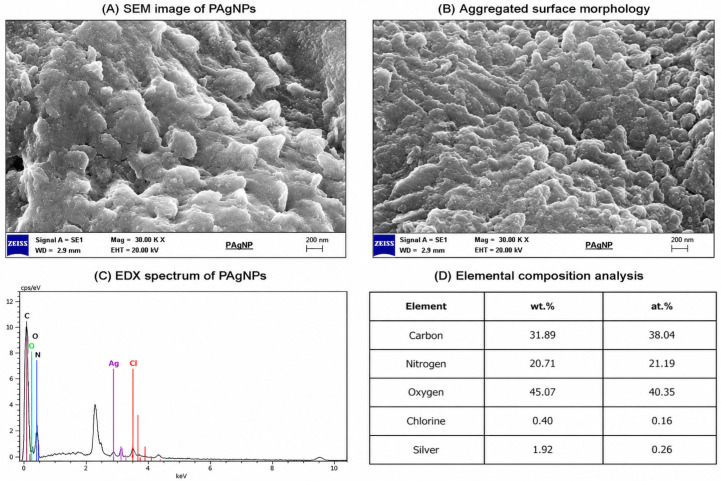
SEM images and EDX analysis of PAgNPs synthesized using *Quercus robur* acorn extract. (**A**,**B**) SEM image showing the aggregated surface morphology of the nanoparticles at higher magnification. (**C**,**D**) EDX spectrum confirming the presence of silver as the major element along with minor signals attributed to residual phytochemicals.

SEM-energy dispersive X-ray (SEM-EDX) spectroscopy was used to confirm the formation of pure silver or silver oxide particles of AgNPs in the elemental composition (Figure 5). SEM analysis revealed densely packed and irregular surface morphologies characterized by significant aggregation of nanoparticles. The observed clustered structures are typical for plant-mediated green synthesis and are likely caused by interparticle interactions, including van der Waals forces and the presence of phytochemical capping agents.

Individual nanoparticles could not be distinctly resolved due to their nanoscale dimensions (20–50 nm) and the micrometer-scale resolution of SEM under the applied conditions. Therefore, SEM analysis primarily provides information on surface morphology and aggregation behavior, whereas precise particle size and shape were determined using TEM analysis.

The apparent discrepancy between SEM and TEM observations arises from differences in sample preparation and imaging scale. While TEM enables visualization of individually dispersed nanoparticles, SEM images represent aggregated clusters formed during drying and deposition of the sample.

Although silver nanoparticles are typically spherical, the irregular structures observed in SEM images may result from aggregation of nanoparticles during the synthesis process. This situation is in agreement with other studies in the literature [32].

In a study in which AgNPs synthesis was carried out using the Salvia spinosa (Sage) plant, it was reported that the sizes of the nanoparticles were in the range of 19–125 nm and their shapes were spherical [33]. According to the results, the silver contents of spherical nanoparticles were determined. It has been stated that the presence of strong signals in the silver region (~2.983 KeV) in EDX analyses is important evidence for the formation of AgNPs [34]. In addition, in various studies, it has been stated that the ~3 KeV optical absorption peak in the formation of AgNPs is due to the surface plasmon resonance of silver nanoparticle extracts [35]. Khalil et al. [36] and Nasir et al. [37] found in the SEM analysis of the AgNPs they synthesized that they have an average size of 20–25 nm and 50 nm, spherical morphology and homogeneous distribution, respectively.

The synthesis of PAgNPs was evidenced by EDX spectra determined between 2 keV and 4 keV, which clearly exhibited a strong spectral signal in the silver region at 3 keV. It was determined that the other peak belonged to chlorine (Cl). The presence of chlorine signals may originate from plant-derived biomolecules or residual compounds associated with the extract, supporting the existence of a phytochemical coating on the nanoparticle surface. It is thought that the Cl^−^ ion seen in EDX analysis originates from plant material (Figure 5B). It is reported that these peaks are associated with residual phytochemicals such as phenolic and flavonoid compounds [38].

Based on SEM and EDX analyses, the synthesized nanoparticles exhibit aggregated morphology and the presence of elemental silver, confirming successful nanoparticle formation [39]. These results are consistent with the structural characterization obtained from other analytical techniques.

The SEM images primarily demonstrate aggregation and surface morphology, while individual nanoparticles could not be clearly resolved due to their nanoscale size (20–50 nm) and clustering behavior. Therefore, detailed particle size analysis was performed using TEM.

#### 2.4.5. X-Ray Diffraction (XRD) Analysis

It has been stated that XRD (X-Ray Diffraction) is used to analyze the size, phase identification and crystal structure of AgNPs synthesized by the green synthesis method and that their sizes can vary between 5 and 40 nm [40]. Samples were analyzed by X-ray diffractometer in the range of 3° ≤ 2θ ≥ 80°.

Bagherzade et al. [41] reported that the XRD spectrum of their obtained AgNPs showed four diffraction bands at 2θ = 38.35, 46.46, 64.75, and 77.62, which are the characteristic Bragg diffraction plans of the face-centered cube (111), (200), (220), and (311). In studies conducted using XRD, the diffractions of AgNPs obtained from *Rosa canina* (Rosehip) fruit were (111), (200), (220), (311) and (222), the crystal size was 19.75 nm and the structure was cubic crystal [41]. The diffractions of AgNPs obtained from *Prunus persica* (Peach) fruit leaves were (111), (200), (220), (311) and (222) and the crystal size was 40 nm [42]. It has been reported that the 2θ values of AgNPs obtained from *Mimusops elengi* (Spanish cherry) fruit are 32.56°, 38.45°, 44.69°, 64.86°, 81.77° and the average crystal size is 43 nm [43]. It was determined that the crystal sizes of AgNPs obtained using *Berberis vulgaris* leaves and roots were 50 nm [44].

**Figure 6:**
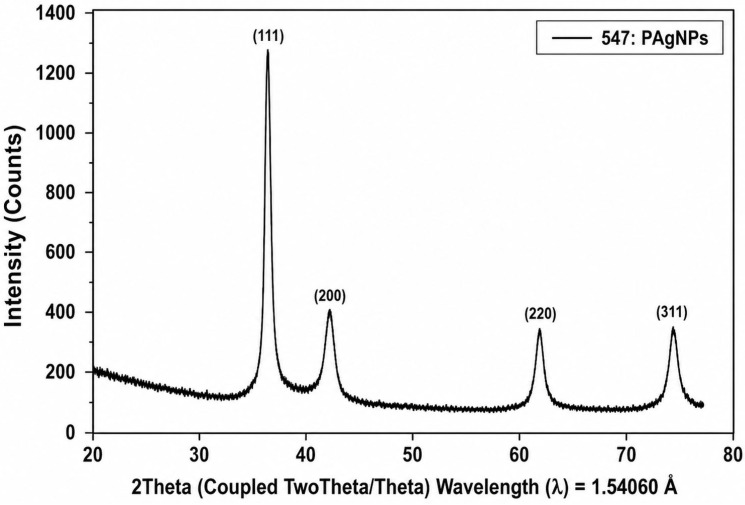
XRD result of PAgNPs sample.

When the X-ray diffraction of the AgNPs obtained according to the XRD results was evaluated, the diameters of the spherical structures of the silver nanoparticles were determined to be 38.10°, 44.05°, 64.52° and 77.46°, respectively, as reflected in the (111), (200), (220) and (311) layers. (Figure 6). It was determined that the formed AgNPs had an elemental (Ag°) and spherical crystal structure.

### 2.5. Biological Activity Tests

#### 2.5.1. Antioxidant Activity Tests of Silver Nanoparticles

Free radical scavenging activities of water extracts and green synthesized PAgNPs were determined according to the method of Blois [45] using DPPH free radical, and Cu (II) reducing power was determined by Apak et al. [46] method and metal binding activity was determined according to the Fe (II)-Ferrozine method of Decker and Welch [47].

#### 2.5.2. DPPH Radical Scavenging Effect of Silver Nanoparticles and Extracts

**Figure 7:**
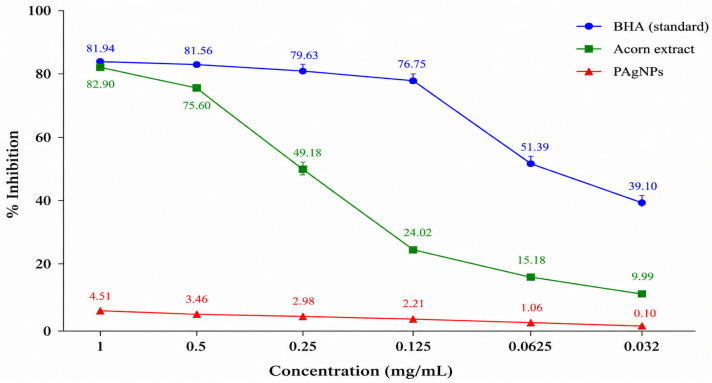
DPPH radical scavenging activity (% inhibition) of acorn extract, PAgNPs and BHA at different concentrations. Values are expressed as mean ± SD (n = 3).

The results are expressed as percentage inhibition and compared with the activity of BHA, a synthetic antioxidant. The DPPH radical scavenging activity increased in a concentration-dependent manner (0.032–1 mg/mL) (Table 4, Figure 7). The acorn extract exhibited a significantly higher radical scavenging activity, reaching 82.90% inhibition at a concentration of 1 mg/mL. In contrast, the activity of PAgNPs was considerably lower, with a maximum inhibition value of 4.51% under the same conditions.

Reddy et al. [48] reported that AgNPs synthesized using Piper longum fruit extract exhibited lower activity than the crude extract and standard antioxidant, with an average inhibition value of approximately 67%. Similarly, another study demonstrated that the DPPH scavenging activity of AgNPs increased with concentration, reaching a maximum value of 55.84% ± 1.31% at the highest tested concentration [49].

The reduced antioxidant activity of PAgNPs in the present study may be attributed to the consumption of phenolic and flavonoid compounds during nanoparticle formation and their subsequent binding onto the nanoparticle surface [50]. Consequently, these bioactive compounds are less available in free form to participate in radical scavenging reactions. Similar findings have been reported by Balkan et al. [51] and Küp et al. [52].

#### 2.5.3. CUPRAC Method

Copper-reducing antioxidant capacity (CUPRAC) was calculated based on the Trolox calibration curve and expressed as mM trolox/mg extract.

Reducing power can be an important factor in a compound’s antioxidant activity [53]. It was observed that the reducing capacity of acorn water extracts and PAgNPs increased with increasing concentration, as did the antioxidant activity. According to the CUPRAC antioxidant test results, the Trolox equivalent of the extracts at 1 mg/mL concentration was determined to be 29.638 ± 0.11 for acorn extract and 7.157 ± 0.15 for PAgNPs (Table 5). Additionally, a linear decrease is observed in Trolox equivalent amounts depending on the decrease in concentration. Zengin et al. [54] reported a value of 208.18 mg TE/g as a result of their study with Sideritisozturkii. These values are similar to previous studies by Sanda et al. [55] and the antioxidant activity of silver nanoparticles was lower than the extract due to the fact that not all phenolic components participated in silver reduction and the presence of other compounds whose concentration increased [56]. However, the antioxidant properties of any pure substance may operate on different mechanisms. In summary, antioxidant compounds can demonstrate their antioxidative properties through different mechanisms such as binding transition metals, degrading peroxides, and radical scavenging [57].

#### 2.5.4. Iron Chelating Activity

As shown in Table 6, the results of metal ion chelating activity show that acorn extract and AgNPs have the ability to reduce ferric (Fe^3+^) to ferrous (Fe^2+^) ion. Removal of the red color of the reaction mixture is due to the reduction of iron ions by plant extracts. Acorn extracts showed higher activity (31.463 ± 0.36 mg EDTA/g extra, 29.756 ± 0.12) than PAgNPs. Similar results were reported by Mahendran and Kumari [58] for *Nothapodytes nimmoniana* (Graham) Mabb., where fruit extracts exhibited higher reduction ability than the synthesized nanoparticles. The iron chelating activity of silver nanoparticles synthesized from *Daphne oleoides* SCHREBER subsp. *oleoides* SCHREBER was reported as 11.73 mg EDTAE/g, whereas the corresponding extract exhibited 37.96 mg EDTAE/g. In similar studies, Djermane et al. [59] reported 8.57 mg EDTAE/g for *Thymelaea hirsuta*, Arika et al. [60] reported 20 mg EDTAE/g for *Gnidia glauca*, and Noman et al. [61] reported 93.1 mg EDTAE/g for *Thymelaea microphylla*. Low values in the metal chelation test can be attributed to the presence of secondary metabolites in low concentrations. Since secondary metabolites are bound with silver, they are not free and therefore low metal chelation values may occur [9].

### 2.6. Determination of Quantitative Antibacterial Activity by Disk Diffusion Method and MIC by Broth Microdilution Method

Velmurugan et al. [62] reported that the synthesized AgNPs had significant antibacterial effects on Gram-negative and Gram-positive bacteria. Antimicrobial, antifungal and anti-inflammatory activities of Quercus extracts have been shown in various studies [63,64,65]. Akkuş et al. [66] determined that different extracts of Strandja oak showed antibacterial effects against *Enterococcus* spp., *S. epidermidis*, *E. coli*, and Methicillin-sensitive *Staphylococcus aureus* strain (Table 7). It is assumed that the antimicrobial activity of AgNPs is closely related to the formation of “pits” in the cell wall of bacteria, leading to increased membrane permeability and cell death [67]. Sondi and Salopek-Sondi [68] stated that the bactericidal effects of silver ions are primarily due to their interaction with the cytoplasm inside the cell. Silver ions appear to penetrate through ion channels without damaging cell membranes, as they denature the ribosome and suppress the expression of enzymes and proteins required for ATP production, which enables the breakdown of the cell [67,68].

In the disk diffusion method, Acorn Soxhlet water extracts were determined against *E. coli*, *S. aureus*, *P. aeruginosa* and *S. mutans*, acorn-boiled water extracts were determined against *E. coli*, *S. aureus*, and *S. mutans*, and PAgNPs was determined against *S. aureus* and *S. mutans*. mutans test microorganisms. When looking at the MIC, it can be seen that both extracts and PAgNPs have an antibacterial effect on the test bacteria that was tested. The highest antimicrobial effect on *S. aureus* was determined as 200 µL/0.026 mg in PAgNPs, and the highest antimicrobial effect on *S. mutans* was determined as 200 µL/0.42 mg in PAgNPs. The highest antimicrobial effect on *E. coli* and *P. aeruginosa* (200 µL/0.055 mg) was detected in Acorn soxhlet water extracts (Table 8). Disk diffusion results showed that PAgNPs were mainly effective against *S. aureus* and *S. mutans*, whereas Soxhlet extract exhibited broader activity, particularly against *E. coli* and *P. aeruginosa*. MIC results confirmed that PAgNPs displayed the strongest activity against *S. aureus* (26 µg/mL), indicating higher sensitivity of Gram-positive bacteria compared to Gram-negative strains.

Acorn Soxhlet water extracts showed a synergistic effect with OFX on *Escherichia coli* and OFX on *Staphylococcus aureus*. It was determined that none of the PAgNPs showed antagonist effects (Table 9).

The predominance of additive interactions suggests that nanoparticles act independently; therefore, AgNPs did not significantly enhance antibiotic diffusion but contributed as a separate antibacterial agent.

The antibacterial activity observed in PAgNPs is primarily associated with the nanoscale properties of silver rather than the direct action of volatile constituents. Silver nanoparticles are known to interact with bacterial membranes, increase permeability and induce intracellular damage through released Ag^+^ ions and reactive oxygen species [50,67,68]. The moderate activity detected particularly against *S. aureus* suggests that nanoparticle surface interactions play a dominant role, while phytochemicals from the extract mainly function as stabilizing agents rather than primary antimicrobial compounds. The absence of strong synergistic enhancement in most combinations further indicates that the activity originates from nanoparticle–cell interaction rather than classical phytochemical antimicrobial mechanisms.

The results of this study suggest several potential applications for PAgNPs. Due to their notable antibacterial activity against *Staphylococcus aureus* (MIC: 26 µg/mL) and their synergistic interaction with ofloxacin, PAgNPs could be considered as potential candidates for combination therapy to reduce antibiotic doses and combat antibiotic-resistant strains. Furthermore, the presence of a natural phytochemical coating (phenolics, flavonoids, and terpenoids) on the nanoparticle surface enhances their biocompatibility, making them suitable for topical biomedical applications such as wound dressings, antibacterial creams, or coatings for medical devices (e.g., catheters and implants). The moderate antioxidant activity of PAgNPs, although lower than the crude extract, may provide additional benefits by reducing oxidative stress in wound environments. However, further in vivo studies and cytotoxicity assessments are required before clinical applications can be recommended.

## 3. Materials and Methods

### 3.1. Collection and Identification of Herbal Materials

Acorns were collected from Afyonkarahisar, Erkmen town. The plant material was taxonomically identified by Prof. Dr. Mustafa Kargıoğlu, Department of Biology, AfyonKocatepe University. A voucher specimen was deposited in the herbarium of Afyon Kocatepe University for future reference. The acorn samples were cut into small pieces and dried in the shade at room temperature.

### 3.2. Acorn Essential Oil Extraction and Essential Oil Analysis by GC-MS

Essential oil was obtained from plant materials by hydrodistillation (HD) using the Clevenger apparatus [69]. Essential oil analysis was performed to determine the phytochemical profile of acorn and to evaluate the potential contribution of volatile constituents to nanoparticle stabilization. Agilent GC-MS device (Agilent Technologies, Santa Clara, CA, USA) analysis conditions are given in Table 10.

### 3.3. Obtaining Plant Extract by Extraction Method

Two types of aqueous extracts were prepared.

(i) Soxhlet extraction was performed using 15 g of dried acorn material and 300 mL of deionized water for phytochemical and antioxidant analysis.
(ii) For nanoparticle synthesis, 10 g of dried acorn was boiled in 100 mL deionized water at 60 °C for 30 min, filtered (Whatman No.1) and used immediately [70].

### 3.4. Determination of Total Flavonoid Content and Total Phenolic Content

The calibration curves were prepared by measuring the absorbance of gallic acid and quercetin standard solutions at different concentrations using a UV–Vis spectrophotometer (Shimadzu UV-1800, Shimadzu Corporation, Kyoto, Japan) at the specified wavelengths. Total flavonoid content in the plant samples to be used was determined according to the AlCl_3_/potassium acetate spectrophotometric method [71,72]. Total phenolic content was determined by the Folin–Ciocalteau method [73,74,75]. Total phenolic and flavonoid content analyses were performed on the same day for both acorn extract and PAgNPs under the same laboratory conditions (room temperature, protected from light). Prior to analysis, all samples were stored in dark bottles at +4 °C to minimize oxidative degradation.

### 3.5. Green Synthesis of Silver Nanoparticles

For the green synthesis of silver nanoparticles, 5 mL of acorn extract was added to 45 mL of 1 mM AgNO_3_ solution under continuous magnetic stirring at room temperature. The reaction mixture was maintained under constant stirring for 60 min, and the pH of the solution was measured as 5.8 ± 0.1 without further adjustment.

The formation of AgNPs was initially indicated by a visible color change from pale yellow to dark brown, suggesting a reduction of Ag^+^ ions. The reaction progress was monitored visually and subsequently confirmed by UV–Vis spectroscopy through the appearance of the characteristic surface plasmon resonance band. The silver nanoparticles synthesized using acorn extract were designated as PAgNPs [72].

The synthesized PAgNPs were used without additional purification in order to preserve the natural phytochemical coating derived from the acorn extract on the nanoparticle surface, in accordance with the green synthesis approach.

### 3.6. Characterization of Synthesized Nanoparticles

Silver nanoparticles synthesized by the green synthesis method were dried in an oven at 110 °C and characterization studies were carried out. The formation of AgNPs was monitored with a UV-1700 PHARMA SPEC brand UV–Vis spectrophotometer (Shimadzu Corporation, Kyoto, Japan). The formation of AgNPs was monitored using a UV-1700 PHARMA SPEC UV–Vis spectrophotometer. UV–Vis measurements were performed using deionized water as the baseline reference. The spectra of both the acorn extract and synthesized AgNPs were recorded after appropriate dilution to avoid saturation effects in the wavelength range of 300–800 nm under identical conditions. A sufficient amount of the dried nanoparticle sample (1:10; NP:KBr) was analyzed by FT-IR (Fourier Transform Infrared) Spectrometer (PerkinElmer Spectrum Two, PerkinElmer Inc., Waltham, MA, USA) by turning it into a pellet with 100 mg potassium bromide (KBr) and placing it in the sample holder. FT-IR spectrum was taken in the range of 4000–400 cm^−1^ with a resolution of 4 cm^−1^. In order to determine the structural characterization and nanoparticle size, analysis was carried out with the JEOL JEM-1400 PLUS brand model TEM (JEOL Ltd., Tokyo, Japan). Particle size distribution was determined by measuring at least 100 particles using ImageJ software version 1.53 (National Institutes of Health, Bethesda, MD, USA). XRD analysis of the samples was performed with a PANalytical Empyrean brand model device with at least 0.1 gr of sample. The particle size distribution was determined by measuring at least 100 particles using ImageJ software (NIH, Bethesda, MD, USA) The crystallite size was calculated using the Debye–Scherrer equation. The samples were imaged by EDX (EDX–Energy Dispersive X-ray Spectroscopy) with a LEO 1430 VP model SEM device (Carl Zeiss SMT Ltd., Oberkochen, Germany) and a RÖNTEC QX2 brand and model XFlash type X-ray detector (Röntec GmbH, Berlin, Germany). For SEM analysis, a small amount of dried nanoparticle sample was gently dispersed onto a conductive carbon tape mounted on an aluminum stub. To improve conductivity and minimize charging effects, the sample surface was coated with a thin layer of gold prior to imaging. SEM observations were carried out under an accelerating voltage of 20 kV.

### 3.7. Biological Activity Tests

#### 3.7.1. Determination of Quantitative Antibacterial Activity of Plant Extracts and Silver Nanoparticles by Disk Diffusion Method

Disk diffusion technique was used for quantitative analysis of the antimicrobial effect of the plant extract and PAgNPs. Standard strains used as test organisms (*Staphylococcus aureus* ATCC 12600, *Escherichia coli* ATCC 352213, *Pseudomonas aeruginosa* ATCC 11778, *Streptococcus mutans* ATCC 25175). Chloramphenicol (C30) was used as a positive control and sterile distilled water was used as a negative control. All antibacterial assays were performed in triplicate. Sterile paper disks (6 mm diameter) were used in the disk diffusion assay. Plates were incubated at 37 °C for 24 h. Inhibition zones were measured in millimeters including disk diameter.

#### 3.7.2. MIC Determination by Broth Microdilution Method

Serial two-fold dilutions were prepared in Mueller-Hinton broth and MIC values were expressed as µg/mL. A standard inoculum of the microorganism (1 × 10^8^ CFU/mL) was prepared and equal amounts of the antimicrobial agent were added to various dilutions. The lowest concentration at which there was no visible turbidity after incubating the media for 24 h at 37 °C was considered the minimum inhibitory concentration (MIC). MIC values were expressed as µg/mL.

#### 3.7.3. Combination Tests

In this study, Vancomycin (VA30) (Glycopeptides), Clindamycin (DA2) (Lincosamides) and Ofloxacin (OFX5) (Fluoroquinolone) antibiotics were used. For combination testing of plant extracts and PAgNPs, they were first plated 5 mm apart on 150 mm diameter Mueller-Hinton agar plates cultured with organisms adjusted to a 0.5 McFarland standard and incubated at 35 ± 2 °C for 16 to 18 h [70,71]. Synergistic interaction was interpreted based on enhancement of inhibition zones at the intersection area compared to individual agents. Antagonism has been demonstrated by interrupting the inhibition zone at the junction of antimicrobials [76,77,78].

### 3.8. Antioxidant Activity Tests of Silver Nanoparticles

The antioxidant activities of water fractions and PAgNPs were evaluated using three complementary in vitro assays, namely DPPH free radical scavenging, CUPRAC reducing power, and metal chelating activity. The DPPH radical scavenging activity was determined according to the method of Blois [46], where samples prepared at concentrations of 2000, 1000, 500, 250, 125, 62.5, 31.25, and 15.63 ppm were added to microplates along with DPPH solution, while ultrapure water served as the control. After incubation for 30 min at room temperature, absorbances were measured at 517 nm, and inhibition (%) was calculated using the equation % inhibition = (A0 − A1)/A0 × 100, where A0 is the absorbance of the control and A1 is that of the sample [79,80]. The CUPRAC assay was performed following the established method [46], in which mixtures of neocuproine and Cu(II) at pH 7.0 were combined with the prepared samples at the same concentration range, along with ammonium acetate buffer (pH 7.0). Following incubation with the Cu(II) reagent for 60 min, absorbance was measured at 450 nm against the blank [47]. The metal (Fe^2+^) chelating activity was assessed by the Fe(II)-Ferrozine method [77], where prepared sample solutions were mixed with Fe(II) solution and ferrozine reagent, and the absorbance was recorded at 562 nm. BHA and GA were used as reference standards in the DPPH and CUPRAC assays, while EDTA served as the standard in the metal chelating activity assay [74].

## 4. Conclusions

The chemical profile of *Quercus robur* acorn essential oil was determined by GC–MS analysis, and green-synthesized silver nanoparticles obtained from acorn extract prepared at 60 °C were characterized together with their biological activities.

- GC–MS analysis identified 27 compounds corresponding to 95.26% of the total composition, with β-caryophyllene (43.1%) as the dominant constituent, followed by α-pinene (21.1%) and α-humulene (8.2%), indicating a terpenoid-rich phytochemical matrix capable of supporting nanoparticle stabilization.
- The reduction in total phenolic and flavonoid contents after nanoparticle formation suggests that these biomolecules were consumed during Ag^+^ reduction and subsequently adsorbed onto nanoparticle surfaces as capping agents.
- Among tested concentrations, 1 mM Ag^+^ provided controlled nucleation and stable PAgNPs formation, demonstrating a concentration-dependent synthesis mechanism.
- Combined UV–Vis, SEM-EDX, TEM, FT-IR and XRD analyses consistently verified the formation of structurally stable and crystalline AgNPs.
- The characteristic surface plasmon resonance peak at 445 nm confirmed successful nanoparticle formation and indicated particle sizes within the expected nanoscale range.
- FT-IR findings revealed that functional groups containing C=C and C=O bonds were primarily involved in the reduction process, supporting the role of plant-derived organic molecules in both reduction and stabilization stages.

Although partial aggregation was observed, the synthesized PAgNPs exhibited sufficient stability for antibacterial testing. Future studies should focus on optimizing synthesis conditions to minimize aggregation and enhance colloidal stability. Biologically synthesized AgNPs obtained from *Quercus robur* acorn extract exhibited measurable antimicrobial and limited antioxidant activity, indicating that phytochemicals mainly functioned as reducing and stabilizing agents rather than remaining as free antioxidant molecules. The study demonstrates that acorn-derived biomolecules can direct nanoparticle formation and influence biological performance, suggesting potential use of such phytogenic nanostructures in antimicrobial material design.

## Figures and Tables

**Table 1:** Acorn essential oil analysis results.

No	Compounds	(RRI ^a^)	(%)
1	β-Caryophyllene	1614	43.10
2	α-Pinene	1023	21.10
3	α-Humulene	1689	8.20
4	Caryophyllene oxide	2017	4.60
5	Bornyl acetate	1593	2.70
6	Sclareol	2756	2.50
7	Phtalic acid	2555	2.00
8	Limonene	1201	1.90
9	γ-Cadinene	1773	1.30
10	α-Copane	1501	1.20
11	Abietratriene	2522	1.10
12	Calamenene	1854	0.90
13	Camphene	1068	0.60
14	β-Pinene	1111	0.50
15	(Z)-β-Farnesene	1670	0.40
16	α-Terpineol	1706	0.40
17	α-Muurolene	1740	0.40
18	Manoyl oxide	2376	0.40
19	Tricosane	2300	0.40
20	α-Cubebene	1465	0.40
21	Hexadecane	1600	0.30
22	Heneicosane	2100	0.30
23	Pentadecane	1500	0.10
24	β-Myrcene	1165	0.10
25	β-Phellandrene	1210	0.10
26	p-Cymene	1276	0.10
27	Cubenol	2074	0.10
	Total identified (%)	95.26	

RRI: Relative Retention Index, ^a^; Relative Retention Index determined using n-alkanes (C8–C20) on an HP-Innowax column.

**Table 2:** Total phenolics and flavonoid substance amounts of acorn extract and PAgNPs (mean ± SD).

Examples	Total Amount of Phenolic Substances (µg GAE/g)	Total Amount of Flavonoid Substances (µg QE/g)
Acorn extract	136.594 ± 0.700	9.522 ± 0.016
PAgNPs	112.000 ± 0.080	7.000 ± 0.011

Values are the mean of three independent analyzes ± standard deviation (n = 3).

**Table 3:** Presence of different functional groups in acorn extract and silver nanoparticle and their binding information.

Wave Number, cm^−1^		Vibration Band	Functional Groups
Acorn Extract	Silver Nanoparticle		
3417	3427	O-H stretch	Alcohols, phenols
2931	2922	C-H stretch	Aliphatic CH bonds
1622	1631	C=C stretch	Alkene,Aromatic C=C bonds
1394	-	C-N	Aromatic Amines
1060	-	C-N,C-O	Aliphatic Amines

**Table 4:** DPPH radical scavenging activity (% inhibition) of acorn extract, PAgNPs and BHA at different concentrations.

Sample	1 mg/mL	0.5 mg/mL	0.25 mg/mL	0.125 mg/mL	0.0625 mg/mL	0.032 mg/mL
BHA (standard)	81.94	81.56	79.63	76.75	51.39	39.10
Acorn extract	82.90	75.60	49.18	24.02	15.18	9.99
PAgNPs	4.51	3.46	2.98	2.21	1.06	0.10

Data are expressed as percentage inhibition values obtained at different concentrations.

**Table 5:** CUPRAC analysis results of acorn extract and PAgNPs.

Concentration (µg mL^−1^)	Acorn Extract	PAgNPs
1000	29.638 ± 0.11	7.157 ± 0.15
500	16.240 ± 0.08	5.351 ± 0.17
250	8.435 ± 0.05	2.833 ± 0.03
125	4.703 ± 0.03	2.620 ± 0.07
62.5	3.111 ± 0.22	2.296 ± 0.14

Values are means ± standard deviation of three independent analyzes (n = 3).

**Table 6:** Metal chelating activities of acorn and PAgNPs.

Concentration (µg mL^−1^)	Acorn	PAgNPs
1000	31.463 ± 0.36	29.756 ± 0.12
500	27.073 ± 0.01	15.365 ± 0.25
250	24.63415 ± 0.45	14.878 ± 0.32
125	6.0971 ± 0.02	11.463 ± 0.41
62.5	1.219 ± 0.00	0.243 ± 0.23

Values are means ± standard deviation of three independent analyzes (n = 3).

**Table 7:** Inhibition zone (IZ) diameters of extracts and PAgNPs against test microorganisms.

Microorganism	PAgNPs (mm)	Soxhlet Extract (mm)	Boiled Extract (mm)	Positive Control (mm)
*E. coli*	–	14 ± 2.44	15 ± 3.24	34 ± 0.83
*S. aureus*	9 ± 2.45	9 ± 1.62	6 ± 0.81	21 ± 0.84
*P. aeruginosa*	–	14 ± 0.80	–	32 ± 1.64
*S. mutans*	7 ± 1.63	13 ± 0.81	8 ± 0.83	8 ± 1.67

PAgNPs: AgNPs obtained using Acorn extract, values represent mean ± SD (n = 3). “–” indicates no inhibition zone.

**Table 8:** Minimum inhibitory concentration (MIC) values of extracts and PAgNPs.

Microorganism	PAgNPs (µg/mL)	Soxhlet Extract (µg/mL)	Boiled Extract (µg/mL)
*E. coli*	0.42	0.055	0.85
*S. aureus*	0.026	1.7	3.4
*P. aeruginosa*	0.42	0.055	–
*S. mutans*	0.42	0.110	0.85

MIC: Minimum inhibition concentration.

**Table 9:** Determination of synergistic effect in acorn samples with antibiotic combination tests.

Amount of Extract in 50 µL (mg)	Microorganism	Antibiotic	Interaction
Acorn Soxhlet (3.4)	*Pseudomonas aeruginosa*	VA	additive
		DA	additive
		OFX	additive
	*Escherichia coli*	VA	additive
		DA	additive
		OFX	synergism
	*Staphylococcus aureus*	VA	additive
		DA	additive
		OFX	synergism
	*Streptococcus mutans*	VA	additive
		DA	additive
		OFX	additive
Acorn boiled water extracts (3.4)	*Pseudomonas aeruginosa*	VA	additive
		DA	additive
		OFX	additive
	*Escherichia coli*	VA	additive
		DA	additive
		OFX	synergism
	*Staphylococcus aureus*	VA	additive
		DA	additive
		OFX	additive
	*Streptococcus mutans*	VA	additive
		DA	additive
		OFX	additive
PAgNPs (0.202)	*Pseudomonas aeruginosa*	VA	additive
		DA	additive
		OFX	additive
	*Escherichia coli*	VA	additive
		DA	additive
		OFX	additive
	*Staphylococcus aureus*	VA	additive
		DA	additive
		OFX	additive
	*Streptococcus mutans*	VA	additive
		DA	additive
		OFX	additive

VA: Vancomycin, DA: Clindamycin, OFX: Ofloxacin, SE: synergism PZ: Positive control.

**Table 10:** GC-MS analysis conditions.

Analysis Features	Details
Analysis Type	Essential Oil Analysis
Analyzing Institution	Anadolu University Plant, Medicine and Scientific Research Center
Device	Agilent Brand GC-MS (Gas Chromatography-Mass Spectrometry) Device
Column	Agilent HP Innowax Column (0.25 mm × 60 m, 0.25 μm film layer thickness)
Carrier Gas	Helium (0.7 mL/min flow rate)
Column Temperature	60 °C, Increase: 4 °C/minute, Maximum: 240 °C
Sample Volume	2 mL
Injection Volume	1 μL
Ionization Voltage	70 eV
Quantitative Determination Method	Peak Area Calculation
Component Identification Method	Comparison Method with Wiley 9-NIST 11 Mass Spectral Database

## Data Availability

The data generated for this work has been included in the manuscript.

## Abbreviations

The following abbreviations are used in this manuscript:

AgNPs	Silver Nanoparticles
PAgNPs	Plant-Mediated Silver Nanoparticles
CUPRAC	Cupric Ion Reducing Antioxidant Capacity
DPPH	2,2-Diphenyl-1-picrylhydrazyl
EDX	Energy Dispersive X-ray Spectroscopy
FTIR	Fourier Transform Infrared Spectroscopy
GC-MS	Gas Chromatography-Mass Spectrometry
HR-TEM	High-Resolution Transmission Electron Microscopy
KBr	Potassium Bromide
MIC	Minimum Inhibitory Concentration
MBC	Minimum Bactericidal Concentration
NPs	Nanoparticles
QE	Quercetin Equivalent
ROS	Reactive Oxygen Species
SEM	Scanning Electron Microscopy
TEM	Transmission Electron Microscopy
UV–Vis	Ultraviolet–Visible Spectroscopy
XRD	X-ray Diffraction
GAE	Gallic Acid Equivalent
